# Investigation of Surface–Liquid Interaction Relationships in Attapulgite Loaded Wet-Spun Polyurethane Composite Fibers Using Multivariate Analysis

**DOI:** 10.3390/polym18141776

**Published:** 2026-07-20

**Authors:** Cansu Aras

**Affiliations:** Department of Textile Engineering, Faculty of Engineering, Bursa Uludag University, Bursa 16059, Türkiye; cansuaras@uludag.edu.tr

**Keywords:** attapulgite, polyurethane fibers, wet spinning, liquid interaction, surface accessibility, functional composite fibers

## Abstract

Attapulgite (ATP)-loaded wet-spun polyurethane (PU) fibers were produced to investigate the effect of ATP on the surface structure and liquid interaction behavior of PU fibers under static immersion. ATP incorporation changed the surface morphology of PU fibers from smooth and compact to rougher and more porous structures, as confirmed by SEM-EDS and BET analyses. ATP incorporation increased BET surface area from 2.236 to 17.144 m^2^/g and the total pore volume from 0.0050 to 0.0755 cm^3^/g. These structural changes promoted water uptake and methylene blue interaction by improving wetting-assisted liquid penetration and dye diffusion through accessible mesoporous pathways. ATP incorporation also improved the thermal and mechanical behavior of the fibers at appropriate loading levels. The onset degradation temperature increased from 252.35 °C for neat PU to 270.53 °C with 3 wt.% ATP loading. The highest tensile strength value of 10.026 MPa was achieved at 1 wt.% ATP loading. Pearson correlation and principal component analyses showed that methylene blue interaction was more closely associated with pore diameter and pore volume than with ATP content alone. The results also indicate that ATP incorporation is an effective strategy for tailoring the pore accessibility, liquid interaction, and structure-dependent performance of wet-spun PU composite fibers.

## 1. Introduction

The interaction between liquids and fibrous materials plays an important role in systems involving mass transport, absorption, and surface accessibility. In porous fibrous structures, liquid penetration and diffusion are mainly affected by surface wettability, pore accessibility, and the connectivity of internal transport pathways. These parameters directly affect moisture management in textiles, exudate transport in wound dressings, permeability in filtration systems, and resin infiltration during composite manufacturing [1,2,3,4]. In these systems, liquid behavior is mainly influenced by fiber surface chemistry, wettability, pore structure, and the accessibility of interconnected pores within the fibrous network. Therefore, controlling the surface and structural properties of fibrous materials is important for improving liquid transport, absorption, and interfacial interaction in fiber-based functional systems.

Polyurethane (PU) is widely used in flexible fiber-based systems because of its elasticity, mechanical durability, chemical stability, and easy processability. Among fiber production methods, wet spinning is particularly suitable for PU fibers because it allows continuous fiber formation under relatively mild processing conditions. Recent studies have shown that using wet-spun PU fibers is a versatile route for producing functional composite fibers, morphology control, and structural design. Zhuang et al. [5] reported on wet-spun carbon black-based elastic conductive fibers and showed that controlling the spinning solution viscosity improved fiber processability, electrical conductivity, and mechanical durability. Wang et al. [6] developed mussel-inspired wet-spun PU conductive fibers using tannic acid and liquid metal, highlighting the potential of wet spinning combined with surface modification to tailor fiber functionality. In another study, Lv et al. [7] fabricated biomimetic helical PU fibers by non-isometric coaxial wet spinning, demonstrating that wet-spinning parameters can be used to regulate fiber architecture and smart textile performance. In addition, solvent–nonsolvent exchange causes phase inversion and enables control over fiber morphology in wet spinning process. This process produces fibers with porous internal structures and interconnected diffusion pathways. These structural features are important in applications involving liquid interaction because pore accessibility and surface wettability affect liquid penetration, transport, and interfacial behavior within fibrous systems. Therefore, wet-spun PU fibers have attracted attention for biomedical materials, filtration systems, wearable electronics, and other applications related to liquid transport and surface interaction [8,9,10]. However, the relatively hydrophobic nature of PU limits its interaction with aqueous media and reduces accessibility to polar liquids. This limitation becomes particularly important in applications requiring efficient liquid transport, dye accessibility, or moisture-related functionality. Although various surface modification strategies have been proposed to improve PU wettability, many of these approaches require additional processing steps or may adversely affect the intrinsic properties of the polymer matrix [11,12].

Attapulgite (ATP) is a naturally occurring magnesium–aluminum silicate clay with a fibrous rod-like structure, high surface area, and hydrophilic character. Because of these properties, ATP has attracted attention as a functional filler for modifying the structural and interfacial properties of polymer systems. In particular, the fibrous morphology and hydroxyl-rich surface of ATP can improve interfacial interaction, increase accessible surface regions, and support the formation of interconnected pathways within polymer matrices [13,14]. Previous studies on PU/ATP composites have shown that ATP incorporation can improve thermal stability, mechanical properties, surface roughness, and moisture-related behavior through improved polymer–filler interaction. Ti et al. [15] reported that hydroxyl groups on the ATP surface formed hydrogen bonding interactions with carbonyl groups in PU, resulting in improved interfacial adhesion and mechanical reinforcement. Similarly, Peng et al. [16] observed that ATP improved the thermal and mechanical behavior of waterborne PU systems, especially at low filler contents where homogeneous dispersion was achieved. Lee et al. [17] also reported that ATP increased surface roughness and moisture absorption capacity, suggesting that it may affect liquid accessibility and diffusion behavior within PU structures.

Most previous studies on PU/ATP systems have focused on films, membranes, or bulk composites, whereas studies on wet-spun monofilament fibers remain limited. In addition, the effect of ATP-induced structural changes on liquid interaction behavior in porous fibrous systems has not been fully clarified. Although improvements in wettability and liquid interaction have been reported, these changes are rarely associated with pore accessibility and diffusion pathways within the fiber structure. Therefore, the relationship between pore-related structural parameters and liquid–dye interaction behavior in ATP-modified wet-spun PU fibers remains poorly understood. The novelty of the present study lies in combining BET-based pore structure analysis, water absorption and methylene blue interaction experiments, Pearson correlation, and principal component analysis to establish the relationship between ATP-induced pore evolution and liquid accessibility, rather than focusing only on mechanical or thermal improvement. Accordingly, this study investigated the effect of ATP incorporation on the structural, surface, and liquid interaction properties of wet-spun PU fibers. Morphological, chemical, thermal, mechanical, water absorption, and pore structure analyses were carried out to evaluate the role of ATP in modifying fiber characteristics. In addition, Pearson correlation analysis and principal component analysis were used to examine the relationship between pore structure, liquid accessibility, and methylene blue interaction behavior.

## 2. Materials and Methods

### 2.1. Materials

Elastollan C95A thermoplastic polyurethane was supplied by BASF GmbH (Lemförde, Germany and used as the polymer matrix. Attapulgite (ATP) clay was kindly supplied by CB Minerals LLC (Larchmont, NY, USA). Dimethylformamide (DMF) and pure water were obtained from Tekkim (Bursa, Turkiye).

### 2.2. Preparation of Spinning Solutions

The PU spinning dopes containing different amounts of attapulgite were prepared in DMF. A neat PU solution without attapulgite was also prepared as the control sample. Initially, ATP dispersions containing 1, 3, and 5 wt.% ATP relative to PU weight were prepared in DMF using mechanical stirring followed by ultrasonication to ensure homogeneous dispersion. These dispersions were then added to PU solutions, and the mixtures were stirred at 80 °C for 24 h until complete dissolution was achieved. The final PU concentration of all solutions was adjusted to 10% (*w*/*v*). The sample codes and compositions of the prepared spinning dopes are presented in Table 1.

### 2.3. Wet Spinning of PU and PU/ATP Composite Fibers

**Figure 1:**
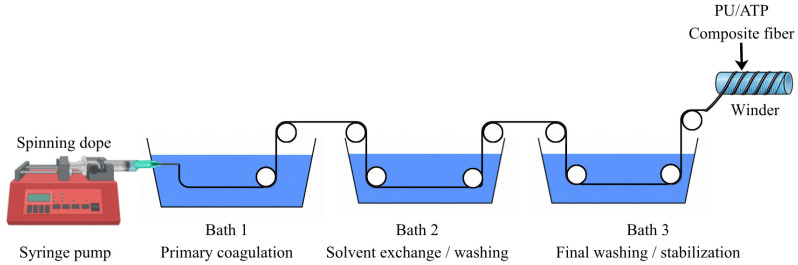
Schematic illustration of the wet-spinning setup used for the fabrication of neat PU and PU/ATP composite fibers.

Wet spinning was carried out using a customized laboratory-scale wet-spinning apparatus. A schematic illustration of the wet-spinning setup used for the fabrication of PU and PU/ATP composite fibers is presented in Figure 1. The prepared spinning dopes were transferred into a syringe and extruded through an 18-gauge needle spinneret (inner diameter: ~0.84 mm) using a syringe pump at a flow rate of 20 mL h^−1^. The extruded fibers sequentially passed through three coagulation baths containing distilled water at the room temperature. Subsequently, the wet-spun fibers were immersed in distilled water for 12 h to facilitate solvent–water exchange and remove residual solvent from the fiber structure. Finally, the fibers were dried in a vacuum oven at 40 °C for 24 h.

### 2.4. Particle Size Analysis

Particle size analysis of ATP was performed by laser diffraction using a Horiba LA-960V2 particle size analyzer (HORIBA Ltd., Kyoto, Japan). The pure water was used as the dispersion medium. ATP powder was gradually dispersed in pure water in the wet measurement cell until the obscuration level recommended by the instrument software was reached. The suspension was agitated and ultrasonicated prior to measurement to reduce particle aggregation. The same refractive index value (*n* = 1.54) was used for all measurements. The particle size distribution was reported on a volume basis, and D10, D50, D90, volume mean diameter, and span index values were calculated. The span index (SI) was calculated using Equation (1).

(1) $$\;SI=\frac{D_{90}-D_{10}}{D_{50}}$$

### 2.5. Morphological, Chemical and Thermal Characterization Analysis

The morphological characteristics of attapulgite, attapulgite-filled polyurethane composite fibers, and neat polyurethane fibers were examined using field emission scanning electron microscopy (FESEM) (GeminiSEM 300, Carl Zeiss Microscopy GmbH, Jena, Germany). Prior to imaging, all samples were sputter-coated with a thin layer of gold to improve surface conductivity. FESEM images were analyzed using ImageJ software (version 1.54, National Institutes of Health, Bethesda, MD, USA). In addition, energy-dispersive spectroscopy (EDS) and elemental mapping analyses were performed to evaluate the elemental composition and surface distribution of ATP within the PU fiber matrix. The elemental composition values obtained from SEM-EDS analysis are provided in the Electronic Supplementary Information (ESI).

The chemical composition of neat PU, ATP, and PU/ATP composite fibers was analyzed by Fourier transform infrared spectroscopy (FTIR) (IRTracer-100, Shimadzu Corporation, Kyoto, Japan)) to evaluate ATP incorporation and its interaction with the PU matrix. Spectra were recorded in the range of 4500–400 cm^−1^ with a resolution of 4 cm^−1^ using 32 scans per sample. The spectra were converted to absorbance mode. Integrated areas of selected characteristic bands were calculated, and normalized band area ratios were determined using relatively stable reference bands.

Thermogravimetric analysis (TGA) was performed using a Shimadzu DTG-60H analyzer (Shimadzu Corporation, Kyoto, Japan) to evaluate the thermal degradation behavior of neat PU and PU/ATP composite fibers. Approximately 5 mg of dried sample was heated from room temperature to 850 °C at a rate of 10 °C min^−1^ under a nitrogen atmosphere. TGA and derivative thermogravimetric (DTG) curves were recorded, and the onset degradation temperature (T_onset_), maximum degradation temperature (T_max_), and residual mass were determined to compare the thermal stability of the fibers.

Differential scanning calorimetry (DSC) analysis was performed using a DSC 25 TA Instruments calorimeter (TA Instruments, New Castle, DE, USA) under a nitrogen atmosphere to investigate the thermal transition behavior of neat PU and PU/ATP composite fibers. The samples were analyzed using a heat–cool–heat cycle. First, the samples were heated from 20 °C to 100 °C at 10 °C min^−1^ and held for 10 min to remove thermal history. They were then cooled from 100 °C to −80 °C at the same rate. In the second heating cycle, the samples were heated from −80 °C to 250 °C at 10 °C min^−1^. The glass transition and thermal transition behavior of the fibers were evaluated from the DSC thermograms, and the thermal transition values were calculated using TA Instruments TRIOS software (version 5.7.0.56, TA Instruments, New Castle, DE, USA). The enthalpy values obtained from the DSC software were initially calculated based on the total sample mass. For the PU/ATP composite fibers, these values were further normalized to the PU mass fraction according to Equation (2).

(2) $${\Delta H}_{PU,normalized}=\frac{{\Delta H}_{measured}}{w_{PU}}$$

where *w*_PU_ is the mass fraction of PU in the composite.

### 2.6. Mechanical Properties of PU Fiber and PU/ATP Composite Fibers

The mechanical properties of neat PU and PU/ATP composite fibers were evaluated by uniaxial tensile testing using a Shimadzu AG-X Plus universal testing machine (Shimadzu Corporation, Kyoto, Japan). The tests were performed with a gauge length of 25 cm, a 5000 N load cell, and a crosshead speed of 250 mm min^−1^ under standard laboratory conditions. Tensile strength, elongation at break, and Young’s modulus were obtained from the stress–strain curves. Young’s modulus was calculated from the linear elastic region between 1–2% strain. All measurements were performed in triplicate, and the results are presented as mean ± standard deviation. Statistical analysis was carried out using Minitab software (version 19.1.1, Minitab, LLC, State College, PA, USA). One-way ANOVA followed by Tukey’s post hoc test was used to determine significant differences between groups at *p* < 0.05.

### 2.7. Porosity and Surface Analysis

Brunauer–Emmett–Teller (BET) analysis was performed using a NOVA 800 surface area analyzer (Anton Paar QuantaTec Inc., Boynton Beach, FL, USA) to evaluate the surface area and pore characteristics of neat PU and PU/ATP composite fibers. Nitrogen adsorption–desorption measurements were carried out at 77.35 K after degassing the samples under vacuum at 40 °C for 12 h. Specific surface area was calculated by the BET method, while pore characteristics were evaluated using the BJH and t-plot methods. Total pore volume was determined at P/P_0_ = 0.99.

### 2.8. Water Absorption and Dye Interaction Analysis

Water absorption analysis was performed to evaluate the liquid uptake behavior of neat PU and PU/ATP composite fibers. The initial dry weight of each sample was recorded before immersion in distilled water for predetermined time intervals. After immersion, excess surface water was removed with filter paper, and the samples were weighed to determine the wet mass. Water absorption was calculated according to Equation (3), where *W*_0_ is the initial dry weight and *W_t_* is the wet weight after immersion. All measurements were performed in triplicate, and the results are presented as mean values.

(3) $$Water\;absorption\;(\%)=\frac{W_{t}-W_{0}}{W_{0}}\times 100$$

Dye interaction analysis was performed using methylene blue solution to evaluate the liquid–dye interaction behavior of neat PU and PU/ATP composite fibers. The samples were immersed in a 5 mg L^−1^ methylene blue solution for predetermined time intervals. After treatment, color measurements were performed using a color spectrophotometer, and the total color difference (Δ*E*) values were calculated using the following Equation (4).

(4) $$\Delta E=\sqrt{{\left(L^{*}-L_{0}^{*}\right)}^{2}+{\left(a^{*}-a_{0}^{*}\right)}^{2}+{\left(b^{*}-b_{0}^{*}\right)}^{2}}$$

where *L*^∗^, *a*^∗^, and *b*^∗^ represent the color coordinates of the dyed samples, while *L*_0_^∗^, *a*_0_^∗^, and *b*_0_^∗^ correspond to the color coordinates of the untreated samples.

### 2.9. Multivariate Analysis

Pearson correlation analysis and principal component analysis (PCA) were performed to evaluate the relationship between ATP-induced structural changes and methylene blue interaction behavior of the fibers. ATP content, BET surface area, pore volume, adsorption pore diameter, water absorption, and Δ*E* values were included in the analysis. Before analysis, all predictor variables were standardized using z-score normalization to eliminate scale differences between parameters, while Δ*E* values were kept in their original form for interpretation. Pearson correlation analysis was used to determine the relationships between variables, and the results were visualized using a correlation heatmap. PCA was performed using the correlation matrix to evaluate the combined effects of structural and surface-related parameters on methylene blue interaction behavior. BET surface area was excluded from the PCA model because of its very high correlation with pore volume (r = 0.99). Therefore, ATP content, pore volume, adsorption pore diameter, water absorption, and Δ*E* were included in the PCA. All analyses and visualizations were performed using OriginPro software (version 9.8.0.200, OriginLab Corporation, Northampton, MA, USA).

## 3. Results and Discussion

### 3.1. Particle Size and Dispersion

**Figure 2:**
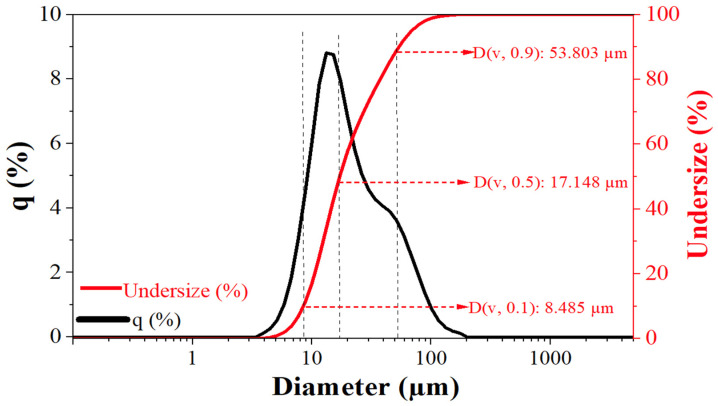
Particle size distribution curve of ATP determined by laser diffraction analysis.

The particle size distribution of attapulgite was determined in an aqueous medium on a volume basis using a laser scattering particle size analyzer (Figure 2). The D10, D50, and D90 values were found to be 8.49 µm, 17.15 µm, and 53.80 µm, respectively, while the mean particle size was 25.21 µm and the mode value was 12.46 µm. These results indicate that ATP particles were mainly distributed within the micrometer range. To evaluate the width of the particle size distribution, the span index was calculated and found to be 2.64. This suggests that the ATP sample had a broad but acceptable particle size distribution. Such a distribution may be beneficial for mineral filler systems used in a PU matrix. Previous studies have reported that fine particles can fill the interstitial spaces between larger particles, thereby increasing packing density, and that this effect is related to the distribution width, expressed as the span value [18]. In addition, studies on PU/ATP systems have shown that the incorporation of ATP can improve properties such as hardness, tensile strength, toughness, and thermal stability when proper dispersion and effective matrix–filler interactions are achieved [19]. Therefore, the obtained particle size profile can be considered a positive preliminary indicator for the use of attapulgite as a functional mineral filler in PU-based composite fibers produced by wet spinning.

### 3.2. Surface Morphology and Elemental Characterization of ATP, PU Fiber, and PU/ATP Composite Fibers

**Figure 3:**
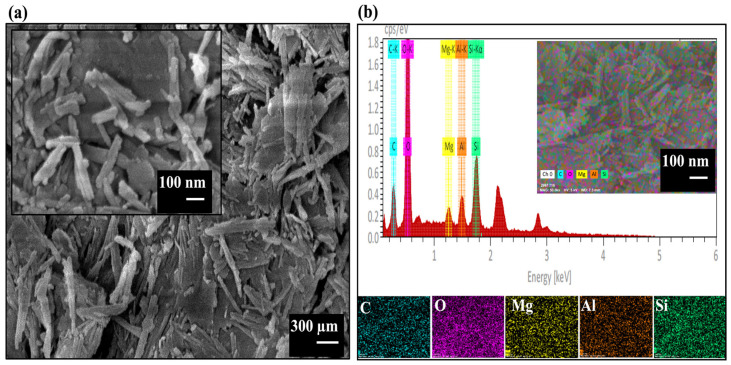
Morphological analysis of ATP, (**a**) SEM images, (**b**) EDS spectrum and elemental mapping images.

SEM imaging, EDS analysis, and elemental mapping were performed to investigate the surface morphology and elemental distribution of ATP, PU fiber, and PU/ATP composite fibers. SEM micrographs of ATP (Figure 3a) revealed the characteristic rod-like fibrous morphology of attapulgite with aggregated bundle-like structures. The particles exhibited relatively high aspect-ratio fibrillar features, which may facilitate interfacial interaction and the formation of interconnected regions within polymer matrices. Similar fibrillar morphologies have previously been reported for natural attapulgite clay minerals [20]. EDS analysis (Figure 3b) confirmed the presence of Mg, Al, Si, and O as the principal elements [21], consistent with the characteristic chemical composition of ATP. Elemental mapping further demonstrated a relatively uniform distribution of Mg, Al, Si, and O throughout the analyzed region, consistent with the characteristic elemental composition of ATP.

**Figure 4:**
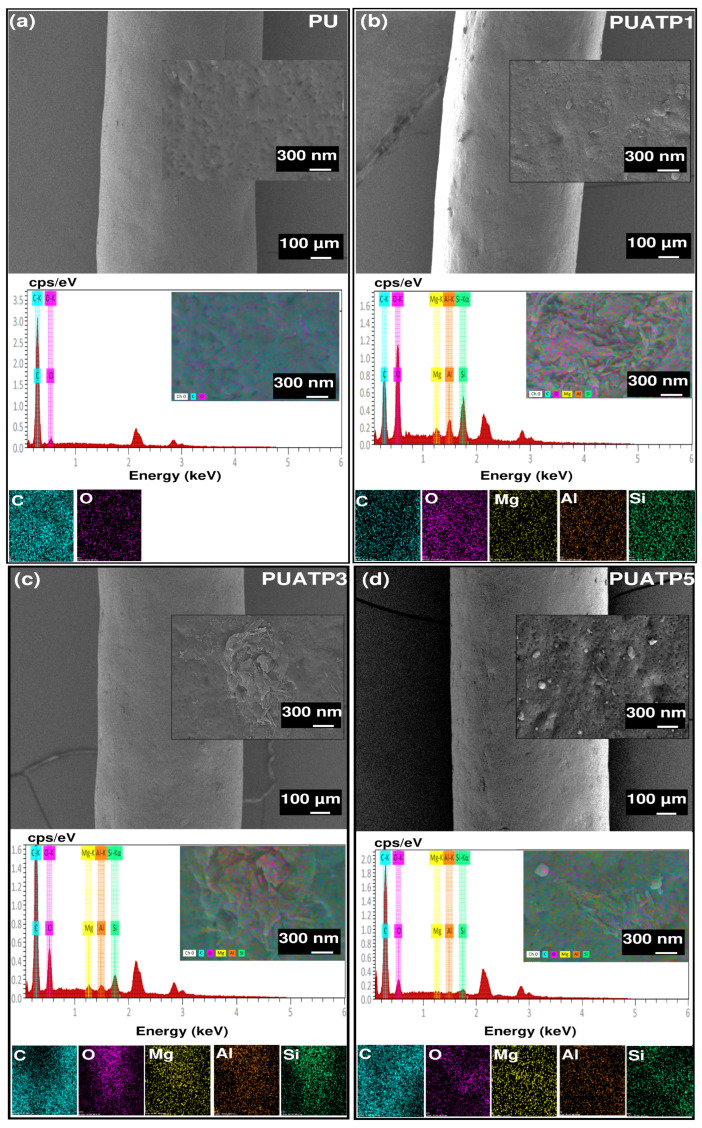
SEM images, EDS spectra, and elemental mapping results of (**a**) PU, (**b**) PUATP1, (**c**) PUATP3, and (**d**) PUATP5 fibers. (*The main SEM images show the fiber surface morphology at higher magnification, whereas the SEM insets provide lower-magnification overview images of wider surface regions to illustrate the general fiber continuity, surface roughness, and local morphological heterogeneity. The EDS spectra and elemental maps provide information on the surface elemental composition and distribution*).

Figure 4 shows the SEM surface morphology, EDS spectra, and elemental mapping results of PU fibers and PU/ATP composite fibers containing different amounts of attapulgite (PUATP1, PUATP3, and PUATP5). The results showed that ATP incorporation altered both the fiber surface morphology and elemental distribution. The neat PU sample (Figure 4a) exhibited a smooth, uniform, and continuous fiber surface without obvious particles or defects. The SEM-EDS elemental composition data provided in Table S1 of the ESI further support the presence of ATP-related elements on the composite fiber surfaces. However, these values should be interpreted as semi-quantitative surface data due to the localized nature of EDS analysis. The EDS spectrum mainly showed only C and O oxygen peaks, consistent with PU matrix. After ATP incorporation, morphological changes were observed depending on ATP contents. PUATP1 sample (Figure 4b) showed slight surface roughness and small particle deposits. Mg, Al, and Si peaks observed in the EDS spectrum confirmed ATP incorporation into the polymer structure, while elemental mapping showed the distribution of these elements along the fiber surface. PUATP3 composite fiber surface (Figure 4c) became rougher, and localized rod-like mineral domains appeared, indicating increased ATP accumulation in some regions. The higher Mg, Al, and Si signal intensities in the EDS spectrum were also consistent with the increased ATP content. In PUATP5 sample (Figure 4d), surface heterogeneity became more pronounced, with nodular protrusions, exposed mineral particles, and disrupted surface continuity. Elemental mapping revealed clustered Mg, Al, and Si regions, indicating reduced dispersion quality at high ATP loading.

### 3.3. FTIR Characterization and Intermolecular Interactions of PU/ATP Composite Fibers

**Figure 5:**
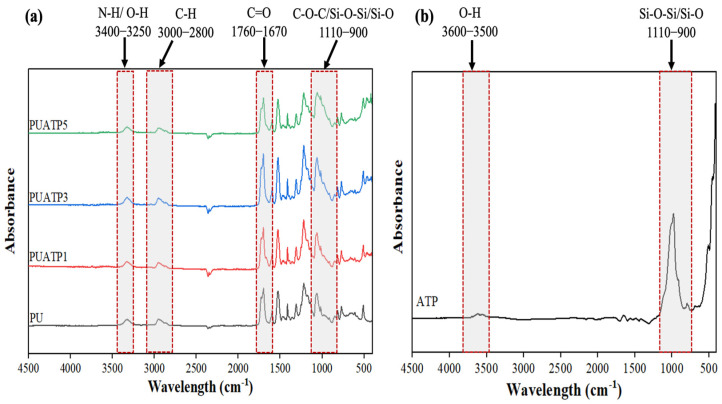
FTIR spectra of (**a**) PU and PU/ATP composite fibers and (**b**) ATP.

**Figure 6:**
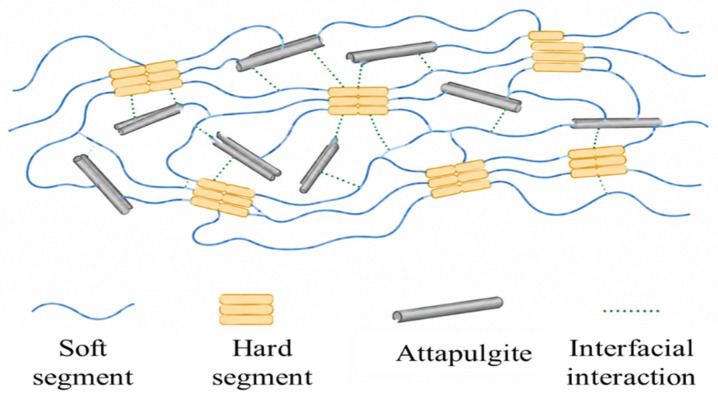
Schematic illustration of the intermolecular interactions between ATP particles and PU chains within the PU/ATP composite fiber structure.

FTIR analysis was performed to investigate ATP incorporation and the intermolecular interactions within the PU matrix. The spectra of neat PU, PU/ATP composite fibers, and pristine ATP are shown in Figure 5. Neat PU exhibited characteristic N-H stretching (3400–3300 cm^−1^), carbonyl (C=O) stretching (1760–1670 cm^−1^), and C-O-C related bands within the 1110–900 cm^−1^ region (Figure 5a) [22,23]. The FTIR spectrum of pristine ATP showed characteristic Si-O and Si-O-Si vibrations in the same region together with a weak -OH stretching band between 3600–3500 cm^−1^ associated with structural water (Figure 5b) [16,24]. After ATP incorporation (Figure 5a), the PU/ATP composite fibers retained the main absorption bands of neat PU, and no new peaks related to covalent bond formation were observed. This indicates that ATP was incorporated mainly through physical dispersion and secondary interfacial interactions (Figure 6) rather than chemical grafting, which is consistent with previous studies [15,25].

To evaluate ATP incorporation, the integrated area of the 1110–900 cm^−1^ region was normalized to the C-H stretching band (3000–2800 cm^−1^), which was selected as an internal reference because of its relative stability among the samples. As given in Table 2, the normalized silicate ratio (Δ*A*_SiOSi_/ΔA_CH_) increased from 2.897 for neat PU to 4.377, 4.220, and 5.394 for PUATP1, PUATP3, and PUATP5, respectively. This result indicates increasing ATP contribution with filler loading and confirms successful incorporation of ATP into the PU matrix. The Δ*A*_NH_/Δ*A*_CH_ ratio was used to monitor changes in the hydrogen-bond-sensitive N-H region. This value increased from 0.889 for neat PU to 0.994 for PUATP5. The increase is mainly attributed to the overlap of ATP-related -OH vibrations with the PU N-H stretching band, together with possible secondary interactions, including hydrogen bonding, between ATP and PU functional groups. In contrast, a decrease in Δ*A*_NH_/Δ*A*_CH_ has been reported by Reddy et al. [25] for organoclay-filled PU systems, suggesting that natural ATP may interact differently because of its distinct surface chemistry. The normalized carbonyl ratio (Δ*A*_C=O_/Δ*A*_CH_) showed a non-linear trend, with the highest value for PUATP1 (3.103), followed by PUATP3 (2.204) and PUATP5 (1.989). This suggests that low ATP loading may provide more efficient polymer–filler contact, most likely because the particles are more uniformly dispersed and present a higher accessible surface area. At higher ATP contents, particle aggregation may reduce the effective interaction between ATP surfaces and PU carbonyl groups.

### 3.4. Thermal Behavior of ATP, PU, and PU/ATP Composite Fibers

**Figure 7:**
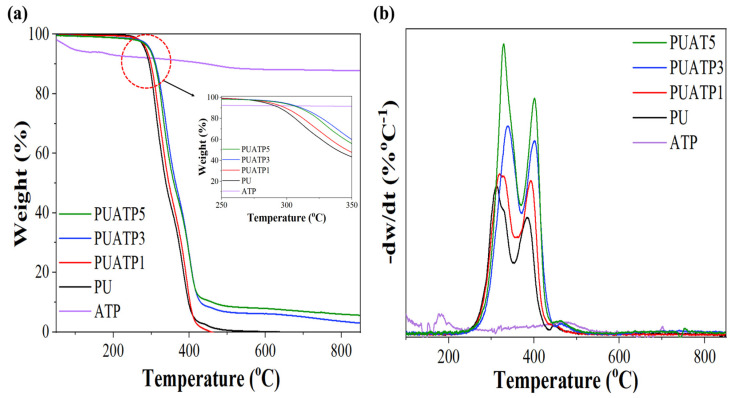
TGA and DTG curves of ATP, PU, and PU/ATP composite fibers; (**a**) TGA curves showing weight retention behavior; and (**b**) DTG curves showing degradation rate peaks. (*The red dashed circle in (**a**) indicates the temperature range enlarged in the inset*).

The thermal behavior of neat PU, ATP, and PU/ATP composite fibers was evaluated by TGA and DTG. Figure 7a shows the TGA curves, while Figure 7b presents the corresponding DTG curves. All samples showed two main degradation stages and the thermal degradation temperatures are given Table 3. The first peak at lower temperature (T_max1_) is assigned to the decomposition of urethane hard segments, whereas the second peak at higher temperature (T_max2_) is related to the degradation of polyol soft segments, which is typical for segmented polyurethane systems [26,27]. Pristine ATP did not show a major degradation step within the decomposition range of PU, confirming the high thermal stability of its structure. A small mass loss around 80 °C was observed, which is attributed to the release of adsorbed and channel water naturally present in attapulgite [28]. Neat PU showed T_onset_, T_max1_, and T_max2_ values of 252.35 °C, 311.44 °C, and 385.55 °C, respectively. After ATP addition, all composite samples retained the two-step degradation profile, but the degradation temperatures shifted depending on filler content. PUATP1 showed increased T_onset_, T_max1_, and T_max2_ values of 265.72 °C, 320.53 °C, and 393.77 °C, respectively, indicating improved thermal resistance after ATP incorporation. PUATP3 exhibited the highest T_onset_ and T_max1_ values of 270.53 °C and 338.71 °C, respectively. This behavior can be attributed to the barrier effect of effectively incorporated ATP particles, whose rod-like structure can hinder heat transfer and prolong the diffusion path of volatile degradation products. In addition, surface hydroxyl groups of ATP may interact with polar urethane groups and locally restrict chain rearrangement (Figure 6), contributing to improved thermal resistance [29]. In contrast, PUATP5 showed lower T_onset_ and T_max1_ values, 261.44 °C and 327.34 °C, respectively, despite its higher ATP content. The sharper and more intense DTG response of PUATP5 indicates a faster degradation rate compared with PUATP3. This suggests that excessive ATP loading did not further improve the thermal barrier effect. At higher ATP loading, reduced dispersion efficiency and possible local structural heterogeneity may limit the effectiveness of ATP as a barrier and facilitate heat transfer and volatile-product diffusion. This observation is also consistent with the SEM in Figure 4, where PUATP5 showed a less uniform surface morphology and possible local heterogeneity at higher ATP loading. Similar findings were reported by Lee et al. [17], where T_max_ values increased up to an optimum ATP content and then decreased at higher loading due to reduced dispersion efficiency. Residual mass at high temperature increased after ATP incorporation because of the non-combustible inorganic structure of ATP. In addition, the remaining silicate phase and possible char formation may reduce heat and mass transfer during the final stage of degradation [16,30].

**Figure 8:**
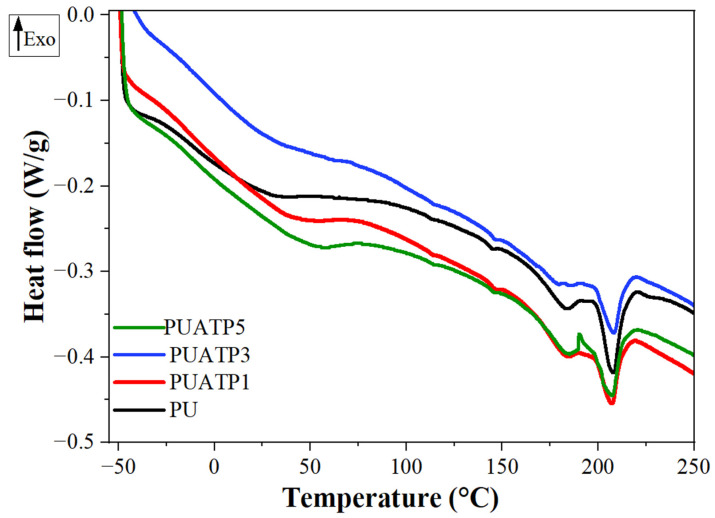
DSC thermograms of neat PU and PU/ATP composite fibers.

The thermal transition behavior of neat PU and PU/ATP composite fibers was evaluated by DSC under a nitrogen atmosphere (Figure 8), and the corresponding transition temperatures are summarized in Table 4. The DSC thermograms revealed a main endothermic transition in the range of approximately 180–220 °C for all PU based samples. In segmented thermoplastic PU systems, this transition is commonly associated with dissociation, relaxation, or partial melting of ordered hard-segment domains rather than the melting of a single homogeneous crystalline phase. Neat PU showed a transition peak at 207.36 °C with an enthalpy change of 8.68 J/g. After ATP incorporation, the peak temperature remained within a narrow range between 206.51 and 207.89 °C, indicating that the fundamental hard-segment transition of the PU matrix was preserved. However, the transition enthalpy changed with filler loading, demonstrating that ATP influenced the extent and organization of thermally active ordered domains. PUATP1 exhibited a peak temperature of 206.51 °C and an enthalpy value of 8.42 J/g, close to neat PU. This suggests that low ATP loading did not significantly disturb hard-segment ordering. PUATP3 showed the lowest enthalpy value with 5.94 J/g, although its peak temperature slightly increased to 207.89 °C. The decrease in enthalpy indicates that fewer or less well-organized hard-segment domains are involved in the transition. Because ATP possesses a high surface area and silanol (Si-OH) groups that can form hydrogen bonding with urethane (-NHCOO) and carbonyl (C=O) groups of PU. Its rod-like morphology can also physically entangle with PU chains and act as junction points within the matrix (Figure 6). This may reduce local chain mobility and strengthen intermolecular interactions in the polyurethane system [29]. This interpretation also agrees with the FTIR results, which indicated secondary polymer–filler interactions rather than new covalent bonding. Such interfacial interactions can suppress local chain rearrangement and reduce the amount of ordered hard-segment domains, explaining the lower transition enthalpy of PUATP3 in DSC. Despite this, PUATP3 showed the highest T_onset_ and T_max1_ values in TGA, indicating that thermal stabilization mainly arose from barrier effects and polymer–filler interactions. At higher loading, PUATP5 showed a peak temperature of 206.87 °C and an enthalpy value of 8.46 J. PUATP5 showed an increase in enthalpy compared to PUATP3. This can be explained by particle aggregation at higher loading, which reduced the contact between ATP and the PU matrix. As a result, fewer polymer chains were restricted. This is consistent with the SEM results, where a less uniform structure and local agglomeration were observed at higher ATP content.

### 3.5. Mechanical Properties of Neat PU and PU/ATP Composite Fibers

**Figure 9:**
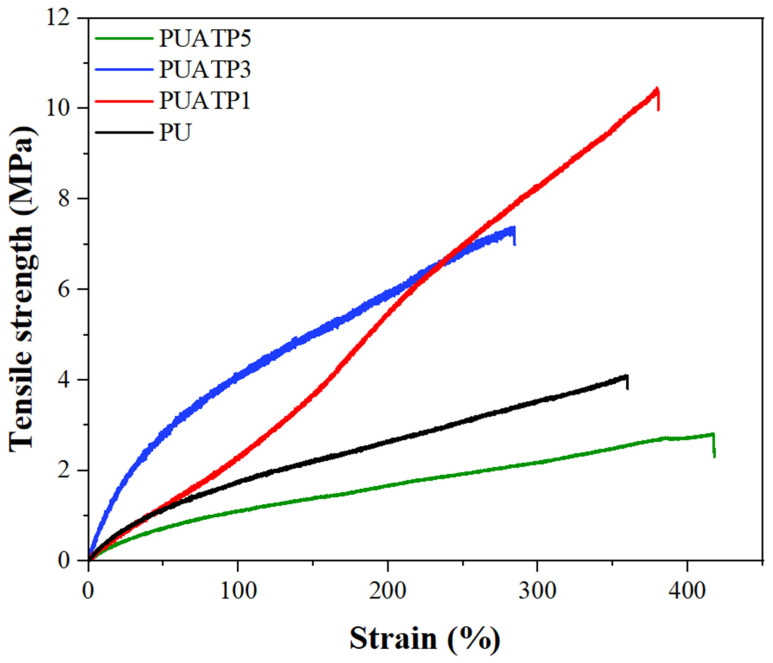
Representative tensile stress–strain curves of neat PU and PU/ATP composite fibers with different ATP contents (1%, 3% and 5% wt.).

Uniaxial tensile testing was performed to evaluate the effect of ATP incorporation on the tensile strength and Young’s modulus of PU fibers. Tensile strength results (Figure 9) showed a statistically significant dependence on ATP loading, according to the Tukey multiple comparison analysis summarized in Table 5. Neat PU exhibited a tensile strength of 4.05 N/mm^2^ and was assigned to “Group C”. With the addition of 1% wt. ATP (PUATP1), tensile strength increased significantly to 10.03 N/mm^2^ and was placed in “Group A”, indicating a statistically significant improvement compared to all other samples (*p* < 0.05). This enhancement is consistent with SEM observations showing good dispersion and with FTIR results indicating effective polymer–filler interactions. At 3% wt. ATP (PUATP3), tensile strength decreased to 7.72 N/mm^2^ and was placed in “Group B”. This value is statistically different from both PUATP1 and neat PU, confirming a moderate but still significant reinforcement effect. The decrease relative to PUATP1 can be associated with the onset of particle aggregation observed in SEM and reduced hard-segment ordering indicated by DSC. At 5% ATP (PUATP5), tensile strength dropped to 2.95 N/mm^2^ and was assigned to “Group C”, the same group as neat PU. This indicates that there is no statistically significant difference between PUATP5 and neat PU (*p* > 0.05). The loss of reinforcement at this loading is attributed to particle aggregation and reduced effective interfacial interaction, as supported by SEM and DSC results.

**Figure 10:**
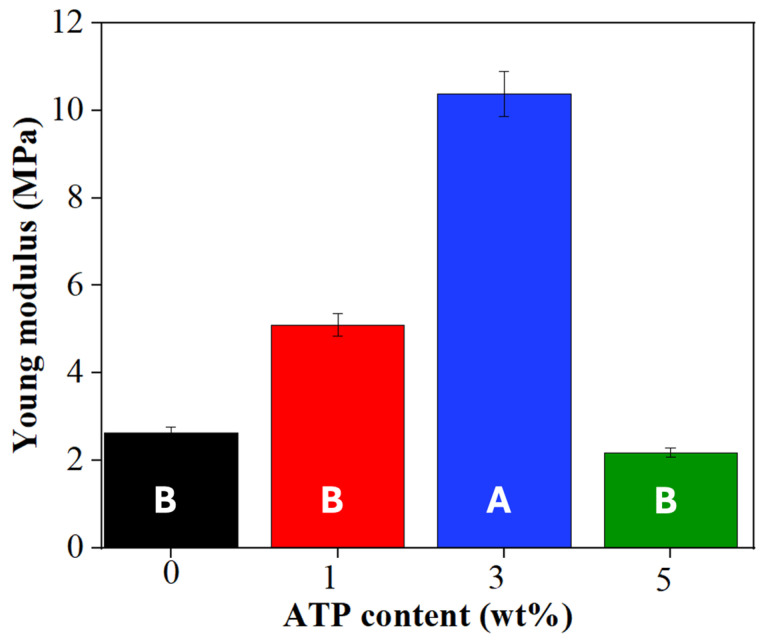
Young’s modulus of neat PU and PU/ATP composite fibers with different ATP contents (*Black, red, blue, and green bars represent PU, PUATP1, PUATP3, and PUATP5, respectively*. *Different letters (A,B) indicate statistically significant differences (p < 0.05)*).

Young’s modulus results (Figure 10) showed that fiber stiffness increased with up to 3% ATP loading and decreased at higher ATP contents. The Tukey multiple comparison results shown in the figure also support this trend. Neat PU showed a modulus of 2.64 MPa and was placed in Group B. In PUATP1, the modulus increased to 5.10 MPa, but the sample remained in the same statistical group, indicating that the increase was not significant. This suggests that 1% ATP was not sufficient to restrict chain mobility. PUATP3 showed the highest modulus value (10.41 MPa) and was placed in Group A, indicating a significant improvement (*p* < 0.05) compared to the other samples. This result is consistent with the DSC findings, where PUATP3 showed the lowest PU mass-normalized enthalpy value of 6.12 J/g PU, and with the TGA results, where it exhibited the highest T_onset_ and T_max1_ values of 270.53 °C and 338.71 °C, respectively. These results suggest that 3 wt.% ATP provided more effective polymer–filler interaction, leading to a stiffer fiber structure, improved thermal stabilization, and more efficient stress transfer. In contrast, at 5 wt.% ATP loading, the modulus decreased to 2.18 MPa and returned to Group B, similar to neat PU. PUATP5 also showed lower tensile strength, while its DSC enthalpy increased to 8.88 J/g PU and its T_onset_ and T_max1_ values decreased to 261.44 °C and 327.34 °C, respectively. This indicates that excessive ATP loading did not further improve stiffness or thermal stability. At higher loading, filler–filler interactions and local structural heterogeneity may reduce the effective interfacial area between ATP and PU, weakening stress transfer and limiting the reinforcing and thermal barrier effects. Therefore, PUATP3 provided a more favorable overall balance among mechanical performance, thermal stability, and polymer–filler interaction, whereas excessive ATP loading caused performance deterioration.

### 3.6. Surface Area and Pore Structure Analysis of PU Fiber and PU/ATP Composite Fibers

**Figure 11:**
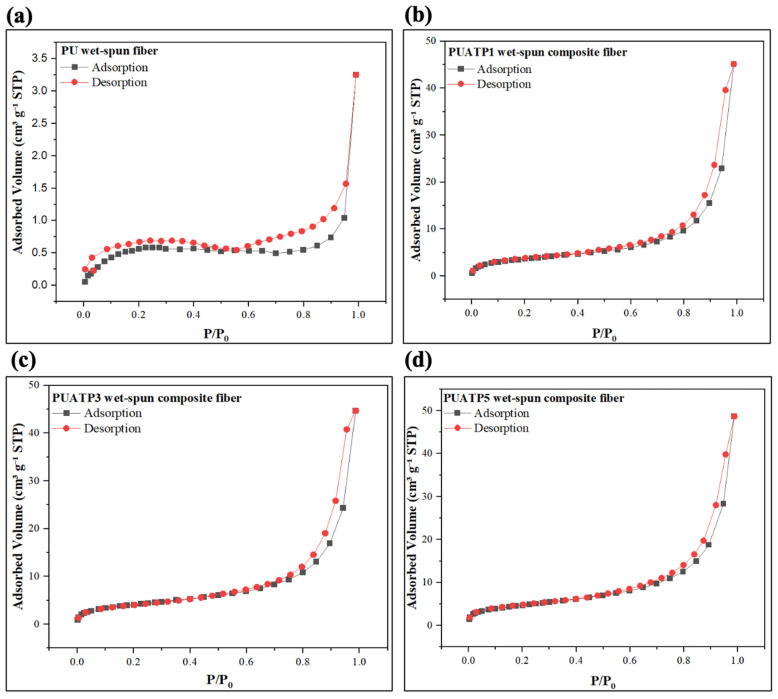
Nitrogen adsorption–desorption isotherms of (**a**) PU, (**b**) PUATP1, (**c**) PUATP3, and (**d**) PUATP5 wet-spun fibers.

The nitrogen adsorption–desorption isotherms of PU and PU/ATP composite fibers are shown in Figure 11, and the calculated BET parameters are summarized in Table 6. All samples exhibited type IV isotherms, indicating predominantly mesoporous structures within the wet-spun monofilament fibers.

Neat PU fibers showed relatively low adsorption capacity with a BET surface area of 2.236 m^2^/g and a total pore volume of 0.0050 cm^3^/g, indicating a compact structure with limited accessible surface regions. In contrast, ATP-containing fibers exhibited significantly higher adsorption capacities. The BET surface area increased to 15.066, 13.520, and 17.144 m^2^/g for PUATP1, PUATP3, and PUATP5, respectively, while the total pore volume increased to 0.069–0.075 cm^3^/g. These results indicate that ATP incorporation increased the accessible porous structure of the PU fibers. Differences in the adsorption–desorption behavior were also observed between neat PU and ATP-containing fibers. Neat PU showed a more pronounced separation between adsorption and desorption branches, particularly at intermediate relative pressure regions (0.4–0.6), indicating less accessible and more heterogeneous internal porous domains. In contrast, ATP-containing fibers showed more continuous adsorption behavior and narrower hysteresis loops, suggesting improved pore accessibility within the fiber structure. The adsorption increase at high relative pressure regions (0.8–1.0) further indicates the presence of accessible mesoporous regions formed during the wet-spinning and coagulation process. BJH analysis also showed changes in pore size behavior after ATP incorporation. Neat PU exhibited similar adsorption and desorption pore diameters (4.319 and 4.914 nm), suggesting a relatively uniform pore structure. In contrast, ATP-containing fibers showed larger and more variable pore diameters. PUATP1 exhibited adsorption and desorption pore diameters of 9.549 and 15.144 nm, respectively, indicating the formation of larger accessible mesoporous regions. PUATP3 showed adsorption and desorption pore diameters of 7.759 and 3.684 nm, while PUATP5 exhibited values of 3.414 and 11.820 nm, respectively. These variations suggest that increasing ATP content affected pore uniformity and resulted in more heterogeneous mesoporous structures within the fibers. The BET results were also consistent with SEM observations. Neat PU fibers exhibited relatively smooth surfaces, while ATP-containing fibers showed rougher and less homogeneous morphologies. Surface irregularities became more visible with increasing ATP content, which likely contributed to the increased nitrogen adsorption behavior of composite fibers. A relationship was also observed between pore structure and mechanical behavior. PUATP1 exhibited both relatively high surface area and higher tensile strength compared to neat PU. In contrast, although PUATP5 showed the highest surface area and pore volume, its mechanical performance decreased. This result suggests that increasing ATP content enhanced accessible porous regions within the fibers, while excessive ATP loading may have negatively affected the structural continuity of the fiber matrix.

### 3.7. Water Absorption and Dye Interaction Behavior of PU Fiber and PU/ATP Composite Fibers

**Figure 12:**
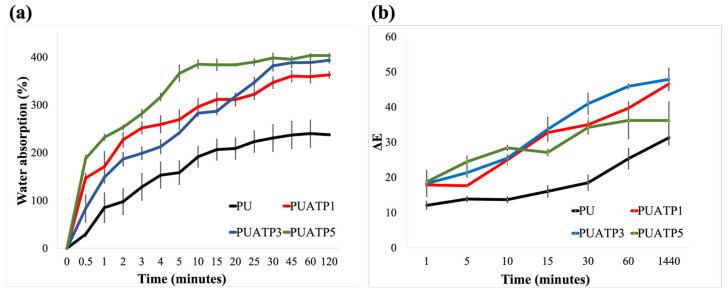
Liquid interaction behavior of neat PU and PU/ATP composite fibers: (**a**) water absorption and (**b**) methylene blue interaction expressed as total color difference (Δ*E*) over immersion time.

Water absorption behavior was evaluated to investigate the effect of ATP incorporation on the liquid uptake capacity and surface accessibility of wet-spun PU fibers under static immersion conditions. The water absorption behavior of neat PU and PU/ATP composite fibers, which was interpreted as an indicator of immersion-driven wetting and liquid penetration into accessible surface and pore regions, is shown in Figure 12a. Neat PU fiber showed the lowest water uptake, reaching about 240% after 120 min. Water absorption increased after ATP addition for all composite fibers. PUATP1 reached about 360% absorption, while PUATP3 and PUATP5 showed the highest values, close to 400%. This increase can be attributed to the hydrophilic nature of ATP and the development of a more accessible porous structure, as supported by the higher BET surface area and pore volume values of the composite fibers.

The methylene blue interaction test was performed to evaluate the accessibility of the fiber surface toward aqueous media. Methylene blue was selected as a model dye because its interaction with porous and hydrophilic surfaces can provide indirect information about liquid diffusion and surface accessibility within the fiber structure. The Δ*E* values increased with immersion time for all samples (Figure 12b). Neat PU showed the lowest color change, increasing from 12.05 at 1 min to 31.25 after 1440 min. ATP-containing fibers showed higher Δ*E* values throughout the test period, with PUATP1 reaching 46.53 and PUATP3 showing the highest value of 47.85 after 1440 min. These results are consistent with the BET and water absorption findings, indicating that ATP incorporation increased the number of accessible regions available for water penetration and dye interaction.

However, the dye interaction behavior cannot be explained by BET surface area alone. Although PUATP5 had a high BET surface area and high water uptake, its Δ*E* value did not further increase compared with PUATP1 and PUATP3. This suggests that methylene blue interaction was governed by the combined effect of surface area, pore volume, pore accessibility, and diffusion pathway continuity rather than by total surface area only. In this case, accessible and interconnected pore regions may facilitate capillary wetting and dye diffusion into the fiber surface and near-surface regions. At higher ATP loading, possible local heterogeneity and reduced interfacial efficiency may limit the accessibility or continuity of these pathways, explaining why PUATP5 did not show a further increase in Δ*E* despite its high surface area.

These findings can be interpreted within a unified structure–property–function relationship. FTIR results indicated that ATP incorporation did not form a new covalent structure, but changed the relative intensity of the N-H, C=O, and Si-O-Si/C-O-C-related bands, suggesting secondary interactions between ATP and the PU matrix. These polymer–filler interactions may influence PU chain arrangement during wet spinning and contribute to pore evolution. This interpretation is supported by the BET results, where ATP incorporation increased the surface area and pore volume of the fibers compared with neat PU. The resulting accessible mesoporous regions enhanced water uptake and methylene blue interaction under static immersion. Therefore, the liquid interaction behavior of PU/ATP fibers was controlled not only by ATP hydrophilicity, but also by the combined effect of polymer–filler interactions, pore evolution, and surface accessibility.

### 3.8. Multivariate Analysis of Structural Parameters and Dye Interaction Behavior

**Figure 13:**
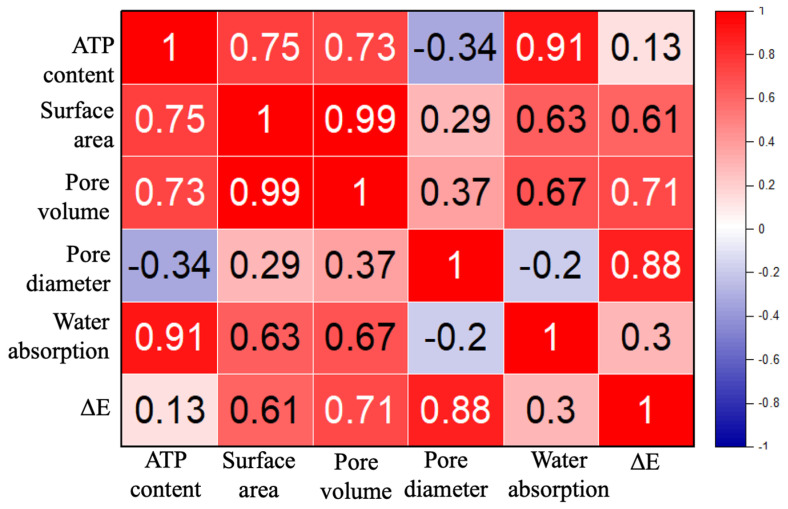
Correlation heatmap of ATP content, BET surface area, pore volume, pore diameter, water absorption, and Δ*E* values of PU fiber and PU/ATP composite fibers.

Pearson correlation analysis and PCA were performed to evaluate the relationship between ATP-induced structural changes and methylene blue interaction behavior. The correlation heatmap (Figure 13) showed that Δ*E* was more strongly related to pore structure parameters than to ATP content alone. Among the investigated variables, adsorption pore diameter showed the highest correlation with Δ*E* (r = 0.88). Pore volume also showed a strong positive correlation with Δ*E* (r = 0.71). These results show that accessible pore pathways and internal pore structure strongly affect dye diffusion within the fibers. BET surface area exhibited a moderate positive correlation with Δ*E* (r = 0.61), indicating that increased surface accessibility also contributed to methylene blue interaction. Water absorption showed a weaker correlation with Δ*E* (r = 0.30), although it was strongly related to ATP content (r = 0.91). This result suggests that liquid uptake alone was not sufficient to explain dye interaction behavior. Instead, pore accessibility and transport pathways played a more important role in methylene blue diffusion. ATP content itself showed only a weak direct correlation with Δ*E* (r = 0.13). However, ATP strongly correlated with BET surface area, pore volume, and water absorption. These findings suggest that ATP influenced dye interaction mainly by changing the pore structure and liquid accessibility of the fibers. A very strong correlation was observed between BET surface area and pore volume (r = 0.99), showing that both parameters originated from similar structural changes caused by ATP incorporation.

**Figure 14:**
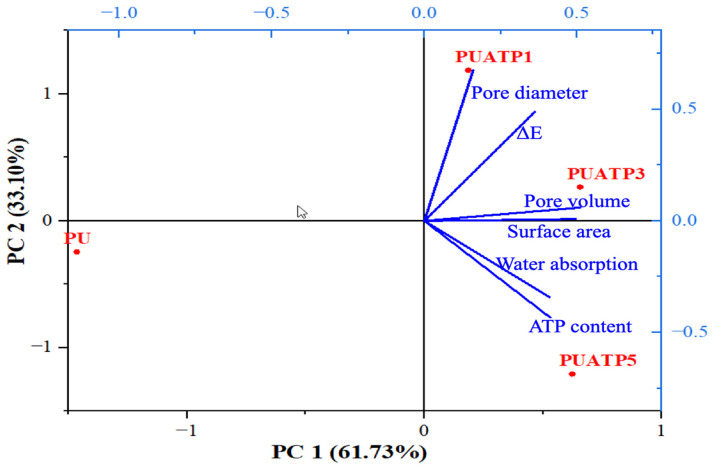
PCA biplot of PU and PU/ATP composite fibers (*PC1 and PC2 explained 94.83% of the total variance*).

The PCA plot showed that the first two principal components explained 94.83% of the total variance (PC1 = 61.73% and PC2 = 33.10%), indicating that most of the differences among the samples could be represented within these two axes (Figure 14). In the PCA plot, the neat PU sample was clearly separated from the ATP-loaded composite fibers. The composite fibers were located on the positive side of PC1, indicating structural changes after ATP incorporation. The PCA biplot also revealed variations in surface area, pore volume, water absorption, and pore diameter among the samples. PUATP3 was positioned closer to the BET surface area and pore volume variables, suggesting a stronger relation to porosity-related properties. The similar orientation of these variables also indicated a positive correlation between surface area and pore volume. In contrast, PUATP5 was more closely associated with water absorption and ATP content, whereas PUATP1 showed a closer relationship with pore diameter. Furthermore, the positioning of Δ*E* near pore diameter and pore volume suggested a relationship between color change and the accessible pore structure of the composite fibers. These findings were consistent with the SEM results. The neat PU fibers showed a smooth and homogeneous surface, whereas ATP-loaded fibers exhibited rougher morphologies. As the ATP content increased, mineral domains became more visible and the fiber surface appeared less uniform. This was more evident in PUATP3 and PUATP5, which also showed stronger association with surface area, pore volume, and water absorption in the PCA.

## 4. Conclusions

ATP-loaded wet-spun PU composite fibers were successfully produced, and the results demonstrated that ATP acts as a structural modifier within the PU fiber matrix. At low and intermediate loadings, ATP improved fiber performance by increasing surface roughness, pore accessibility, and polymer–filler interaction. Compared with neat PU, all ATP-loaded composite fibers showed higher surface area, pore volume, water absorption, and methylene blue interaction, indicating improved liquid accessibility within the fiber structure. PUATP1 exhibited the highest tensile strength, suggesting that low ATP loading provided better reinforcement through more uniform particle distribution. PUATP3 showed the most balanced behavior in terms of thermal stability, stiffness, pore accessibility, and dye interaction. In contrast, PUATP5 reached the highest surface area and water absorption values; however, excessive ATP loading was associated with reduced structural uniformity and lower reinforcing efficiency, resulting in decreased tensile strength and stiffness but increased deformation capacity. Correlation and PCA results further confirmed that pore accessibility, especially pore diameter and pore volume, played a more important role in dye interaction than ATP content alone. These findings indicate that ATP incorporation can be used to tune the structure–property–function relationship of wet-spun PU fibers, particularly for applications requiring controlled surface accessibility and aqueous liquid interaction.

## Figures and Tables

**Table 1:** Sample code of spinning dope solutions.

Sample Code	PU Concentration (% *w*/*v*)	ATP Content (wt.%) *
PU	10	0
PUATP1		1
PUATP3		3
PUATP5		5

* The amounts were calculated based on the weight of PU.

**Table 2:** Normalized ATR-FTIR band area ratios of neat PU and PU/ATP composite fibers calculated using the C-H stretching region (3000–2800 cm^−1^) as the internal reference.

Wavelength/ Sample	1110–900 cm^−1^ (Si-O-Si/Si-O)	1760–1670 cm^−1^ (C=O)	3400–3300 cm^−1^ (N-H/O-H)
	Normalized Value Δ*A*_SiOSi_/Δ*A*_CH_	Normalized Value Δ*A* _C=O_/Δ*A*_CH_	Normalized Value Δ*A*_NH_/Δ*A*_CH_
PU	2.90	2.06	0.89
PUATP1	4.38	3.10	0.93
PUATP3	4.22	2.20	0.92
PUATP5	5.39	1.99	0.99

**Table 3:** Thermal degradation temperatures.

Sample	T_onset_ (°C)	T_max1_ (°C)	T_max2_ (°C)	T_endset_ (°C)
ATP	-	-	-	-
PU	252.35	311.44	385.55	432.40
PUATP1	265.72	320.53	393.77	450.58
PUATP3	270.53	338.71	400.59	432.40
PUATP5	261.44	327.34	400.59	423.31

**Table 4:** Main thermal transition peak and enthalpy values of neat PU and PU/ATP composite fibers.

Sample	Main Transition Peak (°C)	ΔHPU-Normalized (J/g PU)
PU	207.36	8.68
PUATP1	206.51	8.50
PUATP3	207.89	6.12
PUATP5	206.87	8.88

**Table 5:** Tensile strength values of neat PU and PU/ATP composite fibers with corresponding Tukey grouping results.

Sample	Tensile Strength (MPa)	Tukey Group
PU	4.05	C
PUATP1	10.03	A
PUATP3	7.72	B
PUATP5	2.95	C

Different letters (A–C) indicate statistically significant differences between samples (*p* < 0.05).

**Table 6:** BET surface area and pore structure parameters of PU and PU/ATP composite fibers.

Sample	BET Surface Area (m^2^/g)	Total Pore Volume (cm^3^/g)	BJH Adsorption Pore Diameter (nm)	BJH Desorption Pore Diameter (nm)
PU	2.236	0.005	4.319	4.914
PUATP1	15.066	0.069	9.549	15.144
PUATP3	13.520	0.070	7.759	3.684
PUATP5	17.144	0.075	3.414	11.820

## Data Availability

The original contributions presented in this study are included in the article/Supplementary Material. Further inquiries can be directed to the corresponding authors.

## Supplementary Materials

The following supporting information can be downloaded at: https://www.mdpi.com/article/10.3390/polym18141776/s1, Table S1: SEM-EDS elemental composition of pristine ATP, neat PU, and PU/ATP composite fibers.
