# Supplementary material for: Investigation of Surface–Liquid Interaction Relationships in Attapulgite Loaded Wet-Spun Polyurethane Composite Fibers Using Multivariate Analysis
Source: Polymers (Basel). 2026 Jul 20;18(14):1776. doi: 10.3390/polym18141776 (PMC13418589; doi:10.3390/polym18141776)
Supplement: Supplementary file 1 [file polymers-18-01776-s001.zip › polymers-4386559-supplementary.pdf]

Supplementary Information

Table S1. SEM-EDS elemental composition of pristine ATP, neat PU, and PU/ATP composite fibers.

Sample	C (wt.%)	O (wt.%)	Mg (wt.%)	Al (wt.%)	Si (wt.%)	Total (wt.%)
ATP	11.10	47.80	2.62	4.97	33.50	100
PU	100.00	0.00	0.00	0.00	0.00	100
PUATP1	24.74	48.51	0.00	0.39	26.36	100
PUATP3	39.80	31.87	0.00	2.30	26.03	100
PUATP5	71.99	15.41	0.00	0.00	12.60	100
